# Delivery of precision medicine – *Cambridge Prisms: Precision Medicine* webinar event transcript

**DOI:** 10.1017/pcm.2023.21

**Published:** 2023-09-11

**Authors:** Munir Pirmohamed, Matt Prime, Dianne Nicol, Bass Hassan, Harper Vansteenhouse, Anna Dominiczak, Laetitia Beck, Jessica K. Jones

**Affiliations:** 1 University of Liverpool, Liverpool, UK; 2 Roche Diagnostics, Basel, Switzerland; 3 University of Tasmania, Hobart, TAS, Australia; 4 University of Oxford, Oxford, UK; 5 Celldom, Durham, NC, USA; 6 University of Glasgow, Glasgow, UK; 7 Cambridge University Press & Assessment, Cambridge, UK

**Keywords:** precision medicine, COVID-19, digital health, drug repurposing, medical ethics

Precision medicine is a rapidly evolving field that holds the promise of transforming healthcare. Realising this promise requires a comprehensive understanding of the complex healthcare systems in which precision medicine is delivered.

This paper gives the transcript of a recent webinar held for *Cambridge Prisms: Precision Medicine* and explores the challenges and remaining barriers that must be overcome to translate recent medical advancements into real-world clinical practice with the panel discussing a wide range of topics, including the impact of precision medicine on healthcare systems and existing frameworks, therapies and technology, regulatory and ethical considerations and much more.

Co-Chair for this webinar (Figure 1) was *Professor Dame Anna Dominiczak*, the Regius Professor of Medicine at the University of Glasgow in the UK, Chief Scientist of Health in the Scottish Government, and Editor-in-Chief of *Cambridge Prisms: Precision Medicine.* Joining Anna as Co-Chair was *Dr. Harper VanSteenhouse*, who is the Chief Commercial Officer at Celldom, a U.S.-based company that has developed a live single-cell assay platform for screening, development and QC applications in areas including precision medicine.

**Figure 1.**
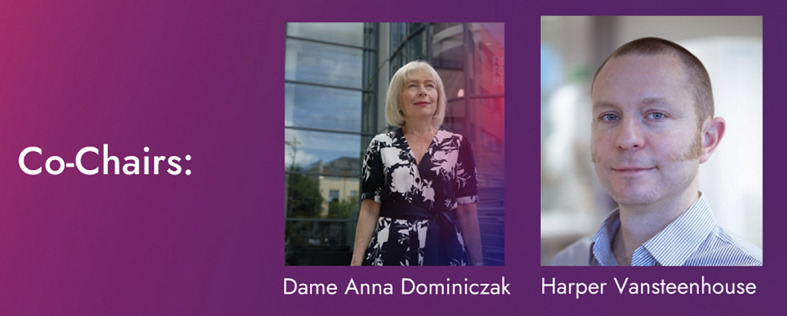
Co-chairs of the delivery of precision medicine webinar event.

The panel (Figure 2) for this event were:

**Figure 2.**
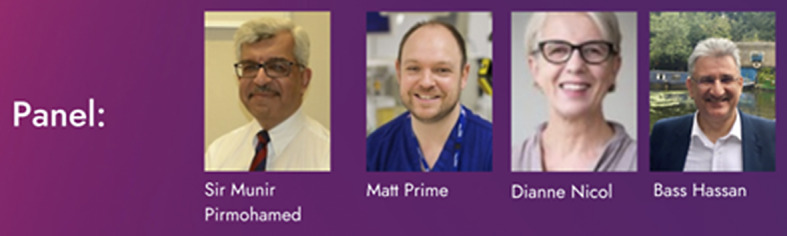
Panellists of the delivery of precision medicine webinar event.

## Professor Sir Munir Pirmohamed, University of Liverpool, UK

Professor Sir Munir Pirmohamed currently holds the David Weatherall Chair of Medicine at Liverpool. He is the Director of the Centre for Drug Safety Sciences, the Director of the Wolfson Centre for Personalised Medicine and the Director of HDR North.

## Dr. Matt Prime, Roche diagnostics, Switzerland

Dr. Matt Prime is the Head of Evidence Generation for Roche Information Solutions based in Basel, Switzerland.

## Professor Dianne Nicol, University of Tasmania, Australia

In 2021, Dianne Nicol retired from her positions as a Distinguished Professor of Law and the Director of the Centre for Law and Genetics at the University of Tasmania in Australia. She continues her involvement in academia as a Distinguished Professor Emerita.

## Professor Bass Hassan, University of Oxford, UK

Professor Bass Hassan is a Professor of Medical Oncology at the Sir William Dunn School of Pathology, University of Oxford, Consultant in Medical Oncology and Clinical Director of Oncology and Haematology at the Oxford University Hospitals NHS Foundation Trust.

View the full recording of the event here: https://www.youtube.com/watch?v=0sfOf1q-ClM&t=115s&pp=ygUZY2FtYnJpZGdlIHByaXNtcyBkZWxpdmVyeQ%3D%3D.

## Begin transcript

### Anna Dominiczak

We would like to discuss with our panellists and with the audience, the opportunities, but also difficulties, potential barriers and blockers to wide-scale implementation of precision medicine into the science clinic industry for all of us. I think the panel has been selected from as diverse experience in precision medicine as we could manage.

### Harper Vansteenhouse

I’ll do a quick introduction so you know who you’re going to be hearing from and as Anna just said we’re eager to have both the prepared questions to start off the discussion but then the audience participation involvement in question. To start off the first speaker I’ll introduce is Sir Munir Pirmohamed who is from the University of Liverpool. Professor Sir Munir Pirmohamed is the David Weatherall Chair of Medicine at the University of Liverpool and has a consultant position at the Liverpool University Hospitals Foundation NHS Trust. He is the Director of the Centre for Drug Safety Sciences, and the Director of the Wolfson Centre for Personalised Medicine. In addition, we have Matt Prime from Roche Diagnostics in Switzerland. Dr. Matt Prime is a global medical director for Roche diagnostic information solutions based in Basel and is also the co-founder of open medical, provider of a leading clinical workflow platform named PathPoint. Bass Hassan is also with us, from the University of Oxford. Professor Bass Hassan’s research is based at the Sir William Dunn School of Pathology at Oxford University. And his translation on clinical practices is in the Oxford University Hospitals NHS Foundation Trust. Dianne Nicol, also on the line with us here, furthest away I think, from the University of Tasmania in Australia. In 2021, Dianne Nicol retired from her positions as a Distinguished Professor of Law and the Director of the Centre for Law and Genetics at the University of Tasmania in Australia. She continues her involvement in academia as a Distinguished Professor Emerita.

I want to thank all the panellists. Welcome. And welcome all the attendees. So, with that we’ll kick off some questions with a few questions from the panel formulated in advance to prepare good discussion in overview and introduction to the topic area.

### Anna Dominiczak

And now we’ll go to the first preprepared general question.

***If you think about how precision medicine is going to behave in the next 5** **to 10 years timeframe, what are you most excited about? What will be the outcomes? And what are also the greatest risks to the successful delivery of precision medicine?***

### Munir Pirmohamed

It is a very broad question and perhaps I can divide it into different areas. The first division is between cancer and non-cancer areas, and the second division would be between ‘niche’ indications and mainstreaming. If we first focus on cancer, clearly this is the poster child for precision medicine. There’s been lots of advance in this area, but I think we can do better. And how can we do better? Well, at the moment, most of the precision medicine in cancer focuses on efficacy to identify new drugs targeting particular somatic mutations which has led to the development of many new medicines. This is understandable – however, we often forget about safety. All these medicines are targeted, but this does not mean that there are no safety issues. Cancer would really be the perfect area of precision medicine if we can focus on both efficacy and safety. Clearly as a clinical pharmacologist, I look at benefit–risk ratio, and the benefits have clearly been improving with the development of novel cancer therapies, but there’s still some risk, and the question that needs to be asked is whether can we further improve the benefit–risk ratio with precision medicines in cancer by also improving safety?

Another area of cancer which is really exciting is the development of more highly personalised therapies, such as cancer vaccines which are coming through using mRNA and other types of technologies. This requires sequencing of the tumour genome to identify neoepitopes which are incorporated into the vaccine, which is highly personalised to that individual, in the hope of improving outcomes including overall survival, and hopefully with reduced problems with side effects as well. Clearly, there remain many challenges with this, with many ongoing trials. There are challenges in terms of regulation and there is a need to define the regulatory pathway.

Now, if I go on to the non-cancer areas, I think the exciting thing that will progress precision medicine is the use different kinds of technologies, not just genomics. And I would hope that in the next 5–10 years, we try to define subtypes of these complex diseases and develop a new taxonomy of disease. We’re still using a taxonomy of disease which dates back from the 19th and 20th centuries, but really, we know that if you have a complex disease such as ulcerative colitis, for example, it’s not just one disease, it’s probably many diseases. Sub-categorisation would help develop a new taxonomy, as is starting to happen in certain areas, such as asthma.

For complex diseases, an area I’m very interested to see how it develops, is the role of polygenic risk scores. There is much interest in the area, but there are some challenges. The most important is the issue of ethnic diversity, where polygenic risk scores may not be portable from one ethnic group to another. This is an area which has attracted a lot of attention and hopefully as we improve the diversity of our participants, the situation will improve. However, irrespective of this, the key question for me is whether polygenic risk scores add any value over and above the clinical risk scores we already have for many diseases.

Another important area is when can we start mainstreaming precision medicine? So far, the focus, particularly in genomics, has largely been on rare diseases and cancer, but these are relatively niche areas restricted to specialists. If we focus on pharmacogenomics, an area of interest to me, if we can mainstream, clinicians (and that means not only doctors but also pharmacists and nurses) can start ordering tests in primary care as well as in secondary care. We do have the evidence to be able to do that, but there are many challenges in implementation, particularly at the technology end in terms of integrating this into the healthcare system. I think one of the other areas which excites me is the development of intelligent decision support systems. No doctor, nurse or pharmacist will have all the knowledge to be able to make sure that the patient in front of them is getting the right medicines that they require. So, we need intelligent decision support systems which can be embedded into the clinical pathway. No clinician is going to have an hour or more to look through the genomics data and other types of omics data while they’re trying to see a patient. We want that information readily available to them in the computer systems while they are seeing the patient, in effect embedding it in the clinical pathway. This is really important. I think there are very interesting new decision support systems coming through which will help us achieve that and enable implementation into clinical practice.

Risks. I think the biggest risk is that this becomes a technology of the rich and exacerbates health and race inequalities. I think Dianne is going to focus on that a bit later. Another risk is the perception that there is competition between precision medicine and public health. I don’t think there is any competition. People talk about ‘me’ medicine and ‘we’ medicine, but I think these are complementary areas and they need to coexist. There is no competition, and I think that both areas can learn from each other. For example, in public health we need to start thinking about precision public health, but in areas of precision medicine we also need to think about it in public health terms as well. So, I think these are areas which can learn from, and complement, each other. One of the biggest risks for precision medicine is the knowledge and skills gap in our healthcare workforce, but also in the public as well, and we need to bridge that gap. I think that needs to start off right through the undergraduate curricula. For example, in the medical curriculum, many medical schools only have 1 day on genomics through the 5-year training programme and that needs to improve. But when you talk to Deans of Medicine they say ‘well you know the curriculum is already fully packed’ and ‘what should I remove?’ I understand this is a challenge, but it is something that needs to be tackled by those in charge of curriculum development to ensure that our doctors, nurses and pharmacists of the future are prepared for these exciting new areas. Obviously, we also need to develop postgraduate education as well in order to bridge the knowledge gap in the existing workforce.

So, I think I’ll stop there. Those are the risks and benefits that I see that are coming through with the developments in precision medicine.

### Anna Dominiczak

Thank you very much. I think I could see heads nodding in the panel. I think we all speak from the same hymn sheet, so could I just push that a little more and ask you:

***We see occasionally discussions, maybe from different parts of biomedical teams, that you can sub stratify too much, that there could be a situation that you’ll be producing management for a single patient perhaps by doing that super precision medicine or whatever you wish to call it, you sub stratify so much that this subgroup is tiny. What do you think? Is this one of the risks?***

### Munir Pirmohamed

Well, you know we are already doing that in some areas. For example, in very rare diseases such as spinal muscular atrophy, highly personalised medicines which are coming through. But I don’t think that will happen in most cases. I think we are not going to be able to undertake ‘super precision’ or ‘very highly personalised medicines’ because stratification will be in subgroups. I think people also worry about the fact that there may be individuals who become orphan individuals where we can’t treat them because there’s no medicine available. But we know with every medicine, even precision medicines, that nothing is 100% predictive. There’s always a degree of doubt in terms of efficacy as well as in terms of safety. What we’re trying to do, in terms of probabilities, is to improve where we are now in terms prediction – at the low end of 10–20%, to increase to 50% or greater. So, I don’t think we’ll have orphan individuals who can’t be treated at all because there is always something in therapeutic armamentarium that we can treat them with, for their diseases. Improving prediction to much higher levels from the current baseline will help improve clinical outcomes.

### Harper Vansteenhouse

I’ll explore a little bit a couple of themes that Munir brought up, and that is the importance of technology into precision medicine. Maybe this is showing my bias, but I think the biggest theme here is the Human Genome Project and our ability to bring genomics and genetic technology to the existing practice of medicine and integrate these things. This has been a common theme all the way through, and I think will be important the future as well.

***In terms of the delivery now of precision medicine, what role can technology play there to improve the delivery but also ensure that there’s accessibility to all patients? And so maybe I’ll have Matt kick us off there. I think that’s right in his area of expertise as well.***

### Matt Prime

I think it’s a really important topic. So, I’m going to talk about how technology supports precision medicine from the perspective of different layers. On the bottom layer we have to think about really high-quality good data management and digital infrastructure. The delivery of precision medicine, precision oncology, is going to require the management of huge volumes of data and the structuring of that data so it’s really important that centres and sites that are doing this have very robust modern infrastructure. And that really incorporates some of the topics that were brought up before around interoperability. We need to have good extensible data models which use the latest interoperability standards like FHIR (Fast Healthcare Interoperability Resource). Once we have good data management, we can think about digitising the clinical workflow, which is an area of challenge. There are lots of tasks that we do as clinicians that could lend themselves to digitalization. Let me give you an example: when we talk about delivery of precision medicine, very often that is centred around a multidisciplinary team (MDT) interaction, which brings together a group of very expensive individuals to make a decision on the case. Now if we’re spending all of our time looking for the right information from within different disparate systems, that’s using up valuable time on administrative tasks when clinicians could be providing direct patient care. Another example is how we coordinate patients during care. You can think about that and in terms of appropriate triage to care and appropriate diagnostic work-up. A lot of that could be automated so that you don’t get to a decision point and then not have the right information.

The reason that digitising the workflow is really important is because we can then start to deploy clinical decision support at the right time during the patient’s journey, and that avoids from a clinical perspective having to go to lots of different systems to get an answer. We need to make this very seamless and very easy. And so, when we think about the types of technologies that are starting to emerge in clinical decision support, again, I’m referring to this along the patient journey, there are technologies emerging now around screening, so screening patients for early identification of disease. When we get into the diagnostic space there’s a huge amount of work around digital pathology, and also there’s a lot more we can do with image analytics in the radiology sphere. And then of course how do we interpret next-generation sequencing data? And this is a hugely voluminous data and can take people a very long time to interpret. If you have human interpretation you start to get disparities between centres, and therefore in care. I think we have to start to standardise that and there are a variety of tools out there that can help with identifying targets and then matching them to the latest therapies.

Now that’s all about the diagnostics and the decision making, but there’s also a really important component of monitoring patients when they’re on that treatment. This links into the patient safety aspect because there are a number of very interesting symptom trackers. They track the patient’s reported outcomes, their experience of care, their symptoms and actually can allow clinicians and clinical teams to intervene much earlier when a patient may be experiencing an adverse event or a side effect for example. That also is then starting to extend care outside of just the walls of the hospital and start to create these kinds of virtual hospitals. I meant that’s the term other people use in a different context, but basically virtualizing care so we have a much more rounded supportive experience for patients. Now all of that, if that’s done in the right way, it can start to build up what I would call a longitudinal patient journey so we can start to really have contextual information around a patient over their cancer journey or for that matter over their lifetime. And so that leads into the benefits of this kind of layer cake of infrastructure workflow and CDS: what can you do with that type of information and data?

I think there’s a huge opportunity in population health, and maybe we can start to think of terms like precision population health, where you can really target interventions to subgroup populations because you’ve got the data to see where they may be experiencing a challenge. But at the same time, it also creates huge opportunities for research and development. And from a research perspective these much richer datasets over a patient’s longitudinal journey are incredibly valuable, but also from a development perspective it allows us to test different digital interventions in these digital environments.

So commonly when you start talking about technology people’s eyes glaze over and they start talking about GDPR and hacks and things like that, so I just thought I’d spend a little bit of time talking about some of those things. If we talk about the security of a solution, all of the solutions, particularly if you think about a UK context, go through a very formal assessment. Products are assessed from a network security perspective, particularly if they’re cloud-based solutions: where does that data live? What’s the protection? And then from a data privacy perspective, again in the UK, we have Caldicott Guardians who are responsible for patient data privacy. In addition, the contracts that are put in place only allow technology providers/manufacturers to do data processing so they’re not really allowed to do anything other than process that data. So in my opinion, people, particularly in the UK context and I’m absolutely sure it applies across many other countries, should be reassured that those assessments are happening. Now we turn to the clinical decision support topic. These are products that would be classed as software as a medical device (SaMD), so they are going to go through an approval process and then after the approval process they’re going to have post-market clinical follow-up. So again, people should be reassured that those solutions are regulated, and they are heavily reviewed.

I thought I’d finish up by just talking about some of the risks that we have here and I’m probably going to talk about it from more of a manufacturer development perspective. I think there’s a huge risk around underinvestment in this space and really underinvestment in the digital infrastructure. And if that is not in place a lot of the stuff we’re talking about, like the implementation of CDS, it just can’t happen. And I would say on that front in the UK it’s really exciting to see the creation of secure data environments, and that’s something that’s just been kicked off. Those secure data environments will give a lot of confidence, but also start to have really robust infrastructure which can be applied for the purposes of research and development. But I also think you’re going to need, or one is going to need, funding for digitalising those workflows and also delivering CDS. And at the moment if you look globally there’s really very little reimbursement for those types of solutions and that’s going to become a problem. It’s however not just about reimbursing the solution. There are sites that deliver precision medicine, and hopefully from an equity perspective that will become all sites, they’re going to have to make a bigger investment in IT support resources so that if there’s a problem with the solution it can be rapidly solved because that will become really important building people’s trust about using these solutions. And then I think also to the point that was made around education around genomics the same applies for digital products. I mean we really need to improve the digital literacy of clinical teams so that they feel like they understand how these products work and what they’re doing, and that will also extend to patients and caregivers. So, for example, a symptom tracker: they feel able to use those types of solutions. All in all, there are good steps being taken, but ultimately to really open up the opportunity and make that available to a much wider group of people, we are going to require investment to rebuild that infrastructure, digitalise workflows and then bring clinical decision support into clinical practice.

### Harper Vansteenhouse

One of the really neat things about technology and software, especially cloud-based software, is it has the ability to scale very widely and therefore democratise a lot of things and give access to groups maybe that aren’t developing as much technology themselves so they can benefit from other developments. But the flip of that, the risk then becomes the data sources become siloed or so specialised. And I think about the differences in terms of how the US *versus* the UK maintain patient data, and you can end up then generating more silos.

***How do we ensure that we’re getting the net benefits of being able to improve healthcare overall by using these tools rather than dividing them further?***

### Matt Prime

If I just say where we are at the moment: if you have a site that’s a large academic medical centre that’s prepared to invest in these technologies, then they are likely to be deploying those at the moment. I think one of the ways you create equity is actually reimbursement because then other sites will see the opportunity and they will be able to make those investments. So, I do think that creating a fair landscape there will definitely help.

In terms of the data silos, it’s difficult to talk about the US because it’s a very complex landscape made-up of multiple different types of health systems and has a very different approach to what they mean by data ownership and things like that. I think if you look at the UK there’s a real opportunity there, particularly around the secure data environment. So, there isn’t really competition in that sense, it’s really about ensuring that those data environments work and that there’s the right resources and you can imagine a sort of Federated model where the secure data environments can start to build greater impact. But, I would say that access to these tools is critical and that is around reimbursement of funding.

### Anna Dominiczak

I think we’ve been all agreeing thus far and let’s try more difficult aspects of precision medicine now. Many recent papers in our new journal and published elsewhere talk about precision medicine being ready for the clinic and it sounds great on paper, but is it happening in practice? And as we already heard, cancer medicine is always at the forefront, but we now see other parts of medicine and public health just behind it.

***Tell us about the barriers to implement precision medicine to the clinic in real time in your clinical practice.***

### Bass Hassan

I think Munir and Matt have covered the main positives. We should look upon what’s happened in the last 5 to 10 years. There is a step change, it is a disruptive technology, and it’s done that. And now the challenge is how to make this commonplace mainstream the inflexion point to scale up. And we’re coming now across all the infrastructure and preparedness of our healthcare system and having all those components in place to really take a technology, be that genomics or otherwise, analyse that information in a reasonable time, provide that information to be communicated to a patient by qualified and trained certified individuals. But that information is understood by the patient, and it is actually effective in improving the outcome for that patient. And getting all those components in line within any healthcare system is actually quite challenging because there are different responsibilities and there isn’t one common ownership goal that can affect it. Now you can try and do that to the NHS in scale and it’s certainly been the case in the academic and specialist centres that progress has been really quite rapid, but it’s still quite piecemeal even within a major hospital in terms of the clinical practise and being up to date and current.

The workforce issue is actually a reality particularly when we are under huge pressure from the workforce perspective right across Northern Europe and the United States that the demand for qualified individuals who have the training, and the expertise is unlimited and we’re not meeting it. So, we have a problem when we come to implementation in how we scale up knowing that we’ve got a relatively limited workforce. And I think Munir’s point about supplementary technologies, artificial intelligence or autopilots, aide memoire prompters, clinical guidelines, automated identification of clinical trials; all of that backroom support is vital to guide clinical decision making.

But, certainly in cancer, one of the big problems is that we’re dealing with two genomes. We’re dealing with the semantic genome and the germline genome. For every person with cancer – 20 are affected, and the outcomes for patients generally, they die, and the mortality is very high. So, we’ve got to be very careful about introducing these technologies and that we don’t over-egg the pudding in terms of what this information is going to do for that patient. And we’re going to be fairly sensible about realising that this sort of dealt with the low-hanging fruit. Now it’s going to get much more complicated, much more technically difficult. Maybe combinations of drugs rather than single agent approaches. Maybe personalised vaccines, although yet to be proven that vaccines in the advanced stage of cancer can be that effective; maybe in the adjuvant setting they’re probably better. But for the patients who are interested in certainty and uncertainty, if you pile on uncertainty for cancer patients, actually you can do a lot of damage and it can be detrimental. While you might be leading the charge in terms of the adoption of personalised medicine, you can do harm. And so, we’ve got to be very structured about how we think about the value of this for the patient, and it does come back down to that question. And certainly, the payers are going to be looking at that issue. They’re also going to be looking at the following issue: if we make this societal investment into our healthcare system, which is a major incremental improvement in virtually every system you can imagine from digital to genomic technologies to training right from medical school or nursing school, it is a huge step change and all players will want to know what the cost-effectiveness this benefit is going to be in terms of their system. So that’s another major challenge.

Coming back to the patients though, the tragedy here, which is lived every day with patients in clinics, is that personalised medicine or genomic assessments particularly are enthusiastically performed but the reality is that very few patients actually benefit. There are patients who may identify a single driver gate of function target for a drug, but you can’t access the drug because you have to go through a compassionate use system and so on. So, there are barriers within the system that even if you do identify something it’s actually very difficult to deliver that in a public system. It may be much easier in a private system. And there are many patients who have gone to go testing hoping that there will be some benefit to them, only to be denied and be told that yes, we’ve tested all these genes, but actually none of them are relevant to you. And that has a huge negative impact on the patient; the sense of isolation, being discarded by the system because, for no fault of anything, they don’t at this moment in time have genetic signals that indicate that they can access a drug. So, I think that those experiences sadly shape the experiences of the healthcare team and that can embed scepticism about whether this strategy is actually universally of benefit to patients. So, we’ve got to be very careful about overcalling the benefits and really focus on the low-hanging fruit that’s already happened and really make the case from the perspective of cost-effectiveness on what we have already. That in a cash-strapped healthcare system right across the world is the key issue. What is the return on the investment and what is the value to the patient? And we still don’t really have large amounts of robust data that really make that case and I think in the next 5 years that’s going to be essential if we’re going to implement our ambitions at scale.

### Anna Dominiczak

Thank you for bringing us into that clinical reality that for every condition the most important is the patient.

### Harper Vansteenhouse

I think Bass’s comments really inspire this next question also, which is around the broader good and how we continue to think about the benefits across society. We’ve had a couple of mentions of precision public health which frankly I don’t hear all that often in precision medicine discussions. I am much more apt to hear about rare and orphan disease successes or n-of-1 studies, which are all very impressive, often displays of really neat science bring to bear to solve a challenging problem, but maybe don’t strike this balance of overall greatest good as well.

***When you think about this balance between the needs of the individual in precision medicine vs the overall benefits and the needs of the broader society and the population, do you think we’re getting that balance right?***

### Dianne Nicol

The focus of my research broadly is on the ethical, legal and social implications of genomics and precision medicine and other new technologies, and there are a host of issues. We’ve already heard some of the regulatory challenges, challenges of personal privacy and so on. But this specific challenge about benefit sharing for society as a whole, I think is one of the really pertinent issues when it comes to precision medicine. Precision, people think of it at the individual level, but I think Harper you’re right we need to start thinking about precision public health. And from the ethical and social perspective the immediate issues are things like equity and distributed justice.

I think this really came to the fore through our experiences with the COVID-19 pandemic. You know we saw the stark inequities in the provision of pandemic-related products, whether it was diagnostics or vaccines or therapists, real disparities whether at the local level, the national level or more particularly at the global level. When you look at the number of people in some countries that have actually been provided with vaccine opportunities, it’s really quite appalling. So, I think I just want to raise three big issues, but you know there are many more that we could consider.

The first of course is cost. As you can see from what’s been said, this has required an enormous effort, an enormous amount of research costs through to development costs across a whole ray of technology areas. And so inevitably when the first products come to the market, they’re going to be enormously expensive because somehow the costs have got to be recovered. And it’s difficult to see how this is going to be made available equitably unless there are government subsidies, and of course these vary so much between countries. We’re already going to see disparity between countries because of the way healthcare systems work.

The second is: which medicines. It is going to be really important to decide which medicines should be the most pertinent. We’ve already heard the particular focus on cancers, which I think is really important, but we need to make sure that this doesn’t just become another tool for wellness for rich people. We need to make sure that this is focused on serious diseases affecting large numbers of people globally. And I just wanted to mention that there is one encouraging thing in the field that I’m particularly interested in at the moment and that is genome editing. And I understand that sickle cell disease is one of the diseases that’s being pursued actively with the genome editing tool, and that is very close to clinical approval. So, I think that’s really important. This is a disease that primarily affects people of African origin. Of course, the next question following on from this is how is this going to be equitably distributed across the African continent? Real challenges.

The third point I just want to briefly mention is: research. So, the research that underpins the precision medicine effort is basically reliant on large genomic databases and there are many large genomic databases that are being shared globally. But we know from quite a bit of literature now that, predominantly, the sources of that genomic data are primarily from people of northern European origin. So, this is again going to create problems because we’ve not got the research dataset from other people globally. One of my roles is I’m co-lead of the Regulatory and Ethics working group of the Genomic Health, the global alliance for genomics and health, and one of the key themes we’re looking at is how to better ensure this diversity in datasets. So, there is awareness of these problems, whether its cost or which medicines or research or a whole array of other issues. I don’t have immediate answers, but I do think these are all issues that we all need to consider very deeply.

### Munir Pirmohamed

This is also one of the areas that I’ve been working in the pharmacogenomics area. Sometimes when you have particular predictors of drug efficacy or safety, the minor allele frequency varies according to different ethnic groups. So, if you then develop a test, it’s often just applicable to one particular population. For example, I developed an algorithm for warfarin dosing but that was mainly derived from European ancestry populations, so when I implemented this in UK clinics, I couldn’t use it in Indian or African population. A challenge has been to ensure that individuals from different ethnic minorities take part in research, so that we can develop algorithms that are more widely applicable. In order to circumvent this, we have been working in African countries so that we can improve anticoagulation there, but also learn from there and bring it back to the UK. These bi-directional interactions therefore help in improving capacity and capability in under-resourced countries, but also help in reducing health and race inequalities. It is important that we don’t just work in the rich countries like the UK, but we also undertake work in other countries and increase capacity and capability in those countries as well to make sure that we have the equity in terms of precision medicine.

***Do you anticipate precision medicine to be still genetics focused in a 5–10 years timeframe? I think all of our panellists think about this quite a bit and mentioned it from a lot of different areas. Who wants to jump in first? We’ll probably give you a couple of quick answers instead of several.***

### Anna Dominiczak

Harper, maybe I can start by referring colleagues and the audience to our published new definition of precision medicine in our journal. It’s online and I know clearly genetics and genomics are hugely important but as we heard throughout this discussion there are so many other things; it is not just genetics and genomics. And I think already, if you look at our definition, everything else: the data, the old technology that Matt referred to and all those layers of technology, are absolutely essential. So having just genomic databases will not deliver to the clinic. And I think it’s hugely important. So please read and adopt the new worldwide definition of precision medicines from our journal.

### Munir Pirmohamed

I think that we do need to move beyond genomics. Genomics is very important, and it will always be very important but there are new things coming through in other omics technologies and in artificial intelligence which we’ll need to research and integrate into clinical practice. One of the areas which I think would be a quick win is to utilise the data we collect on our smartphones and wearables. At present, I collect a lot of data on my phone, such as the number of steps per day and my stride length *etc.* Nobody uses that, but I would be happy to share that, with appropriate safeguards, so that people can then start looking at such data, not only from me, but from millions and millions of other people. This would provide exciting data on how can affect outcomes. The ultimate aim would be to integrate such data with omics technologies that are already with us, and with other technologies which are being developed, into multimodal algorithms.

### Bass Hassan

Maybe I can interject just a little bit to cover the cancer bit. There’s no question that research-wise the challenges are always going to be at the protein level and the cellular level. And with the new technologies available for protein assays going beyond simple mass spectrometry and the ability of us now to be able to modify proteins/the abundance of proteins as a therapeutic option, it allows us to look at the next level of polymers within a cell and how we might target and disrupt those. Particularly the way that proteins lock together and can be unlocked by novel drugs that you wouldn’t have identified simply by sequencing DNA or RNA. So, the next revolution if you like is going to be about how we unpack the cellular disruption that happens in cancers beyond the genome.

### Harper Vansteenhouse

A question around COVID-19. This was obviously a salient point and we’re all trying to take learnings from this really tragic event that happened across the world and make the most of it.

***What advantages do you think, in the digital solutions into clinical practice, have we gained by going through this experience with COVID-19?***

### Matt Prime

I would say COVID-19 completely changed the landscape for digital health solutions and hugely accelerated it. And I think, particularly if you think about again more infrastructure workflow type of products like Telehealth, I mean it enabled clinical practise to continue. So I work with one hospital in the United States where we support multidisciplinary teams and they were told in the morning: ‘look we’re going to stop all multidisciplinary tumour boards because it’s not safe for you to be in the same room’ and the teams said ‘well actually what we could do is just scale a solution that we had in development’ which they did and they managed to continue to have cancer MDTs for the whole of the COVID-19 pandemic. So, I think solutions are really able to maintain care and I think that people build confidence. I think what we need to do now is work out how we sustain that because then it was a case of ‘look we have to do this to maintain care’ and now we have to think about how we sustain the change that we’ve created.

### Harper Vansteenhouse

***Can patient preferences be incorporated and patient values especially into the decision making process around precision medicine?***

### Dianne Nicol

I think that is a really important question. I hope it wouldn’t create a difficulty in the sense that I think patient preferences and values should always be incorporated into decision making about their treatment, that this is a collaboration between the clinician and the patient, and their values must be incorporated in every decision about them. But it’s been pointed out a few times that one of the challenges in this field is the lack of educational opportunities regarding genomics, regarding IT and so on. And I think we all need to get more genomics literate, more IT literate so that the patients have the capacity to understand what’s being offered so that my values and my views are informed.

### Matt Prime

I think there’s another opportunity for technology to help capture some of those preferences and information before a decision is made. And I think perhaps as clinicians we think that we’re sharing that information and we think that the patients and their caregivers are taking it on, and this has been a very open conversation, but we don’t know whether they’re really understanding some of the information. And this information is really complex and it’s going to take a lot of time for people to understand it. And again, then there are opportunities for technology to really help people understand this and really act as educational tools. So again, I think it’s an area technology can really support on.

### Harper Vansteenhouse

***In your view, what is the importance of the gut microbiome in the context of precision medicine?***

### Munir Pirmohamed

I think this is extremely important, but actually a very under-researched area. We need to do much more work on this, not only in terms of how the gut microbiome predisposes to complex diseases, but also in terms of prognosis associated with different diseases. In the area that I work in, in pharmacology, there’s an emerging field of pharmacomicrobiomics. There are two broad issues that need tackling: (i) how do drugs affect the microbiome and in particular could they affect it in an adverse way to change the trajectory of your disease; and (ii) how does a microbiome affect the drugs. Because the number of cells constituting the microbiome is far greater than the number of cells that constitute the human body, these microbiota may metabolise drugs in different ways which may affect the efficacy and safety of drugs. So, the area of pharmacomicrobiomics will become more popular, as much more work needs to be done in that area.

### Bass Hassan

I can give you another specific example where actually this is quite robust now in use of checkpoint inhibitors targeting PD1, PDL1, CTLA4, particularly in patients with advanced high tumour mutation burden, mutation rates, such as melanoma. So, treatment with these checkpoint inhibitors can reactivate your own T cells, your own immune system, to attack the tumour and recognise it as non-self. Well, it turns out that your microbiota in your intestines could make a huge difference in terms of your ability to respond to that intervention, and it’s setting the immunological context for those therapies. So, there’s actually quite a lot of work being done about that and it’s probably going to reach the point that we’ll be modifying the microbiota in patients before we treat them.

### Anna Dominiczak

And I think it’s time for me to thank our panellists for great contributions and thank my co-chair Harper. I would like also to thank the team from Cambridge University Press for organising this. And there will be more webinars, there will be more questions answered, so please look out for future webinars; there will be a series of those. And I think what I heard is that this is a great area both for researchers and clinicians, and the most important part is clearly to talk to patients, Dianne, and to listen to patients. And when I do this, I hear patients and Bass I take into account what you said that we shouldn’t give false hope with our enthusiasm. I agree with this, but the patients generally would like medicine to move and improve. And what I hear from patients is that they would like to participate in clinical trials of new methods and new activities. Matt, that they want to use technology to help the future of medicine. And I think, probably the most important bit we heard is: precision public health. And I would like us in the future to talk more, perhaps with the same panellists because you are all hugely invested in this topic.

## End transcript

View the full recording of the event here: https://www.youtube.com/watch?v=0sfOf1q-ClM&t=115s&pp=ygUZY2FtYnJpZGdlIHByaXNtcyBkZWxpdmVyeQ%3D%3D.

