# Supplementary material for: Delivery of precision medicine – Cambridge Prisms: Precision Medicine webinar event transcript
Source: Camb Prism Precis Med. 2023 Sep 11;1:e31. doi: 10.1017/pcm.2023.21 (PMC10953739; doi:10.1017/pcm.2023.21)
Supplement: Peer review history

# Editor report: Recommendation: Delivery of Precision Medicine - Cambridge Prisms: Precision Medicine Webinar Event Transcript — R0/PR2

**DOI:** 10.1017/pcm.2023.21.pr2

**Affiliations:** Editorial Hub, United Kingdom of Great Britain and Northern Ireland

No accompanying comment.

# Editor report: Decision: Delivery of Precision Medicine - Cambridge Prisms: Precision Medicine Webinar Event Transcript — R0/PR3

**DOI:** 10.1017/pcm.2023.21.pr3

**Affiliations:** Institute of Cardiovascular & Medical Sciences, University of Glasgow College of Medical Veterinary and Life Sciences, United Kingdom of Great Britain and Northern Ireland

No accompanying comment.
